# Our Robots, Our Team: Robot Anthropomorphism Moderates Group Effects in Human–Robot Teams

**DOI:** 10.3389/fpsyg.2020.01275

**Published:** 2020-07-14

**Authors:** Marlena R. Fraune

**Affiliations:** Intergroup Human-Robot Interaction (iHRI) Lab, Department of Psychology, New Mexico State University, Las Cruces, NM, United States

**Keywords:** social robotics, group effects, anthropomorphism, morality, human-robot interaction

## Abstract

Past research indicates that people favor, and behave more morally toward, human ingroup than outgroup members. People showed a similar pattern for responses toward robots. However, participants favored ingroup humans more than ingroup robots. In this study, I examine if robot anthropomorphism can decrease differences between humans and robots on ingroup favoritism. This paper presents a 2 × 2 × 2 mixed-design experimental study with participants (*N* = 81) competing on teams of humans and robots. I examined how people morally behaved toward and perceived players depending on players’ Group Membership (ingroup, outgroup), Agent Type (human, robot), and Robot Anthropomorphism (anthropomorphic, mechanomorphic). Results replicated prior findings that participants favored the ingroup over the outgroup and humans over robots—to the extent that they favored ingroup robots over outgroup humans. This paper also includes novel results indicating that patterns of responses toward humans were more closely mirrored by anthropomorphic than mechanomorphic robots.

## Introduction

Robots are becoming increasingly prevalent, not only behind the scenes but also as members of human teams. For example, military teams work with bomb-diffusing robots (Carpenter, 2016), factory workers with “social” industrial robots (Sauppé and Mutlu, 2015), and eldercare facilities with companion robots (Wada et al., 2005; Wada and Shibata, 2007; Chang and Šabanović, 2015). Such teaming is critical for advancing our society, because humans and robots have different skillsets, which can complement each other’s expertise to enhance team outcomes (Kahneman and Klein, 2009; Chen and Barnes, 2014; Bradshaw et al., 2017). To best implement human–robot teaming, scholars need guidelines for how human–robot interaction (HRI) typically plays out, so they can plan for typical HRI paradigms.

**FIGURE 1:**
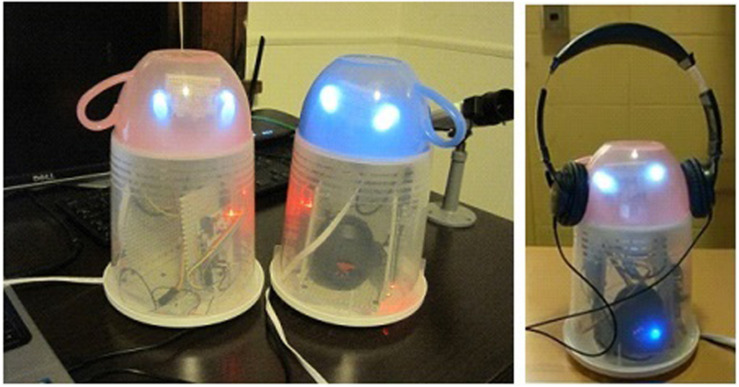
Mugbot robots **(left)**; mugbot with headphones **(right)**. Reproduced with permission from Fraune et al. (2017b).

One strong effect in HRI is that participants favor their ingroup (i.e., teammates) over the outgroup (i.e., opponents), regardless of whether the agents are humans or robots. In several previous HRI studies, participants even assigned “painful” noise blasts to outgroup humans to spare ingroup robots (Fraune et al., 2017b). This was replicated in the United States (Fraune et al., 2020). However, across studies, participants still favored ingroup humans over ingroup robots, though they did not differentiate outgroup humans and outgroup robots. Perhaps most surprisingly, these findings occurred despite that the robots had only a “minimally social” appearance (i.e., a pair of eyes, a head; Figure 1; Matsumoto et al., 2005, 2006), and participants did not view them as particularly anthropomorphic (i.e., having humanlike traits; Fraune et al., 2020).

In this paper, I examine if increased robot anthropomorphism results in more similar ingroup favoritism toward robots like toward humans. I also seek to answer the specific questions: Must robots have some anthropomorphic appearance for people to favor robot teammates over human opponents, or would they do the same with mechanomorphic robots (i.e., robots with machine-like traits)? Further, if the robots had more anthropomorphic characteristics, would people no longer favor ingroup humans over ingroup robots?

To answer these questions, participants entered the lab four at a time and were placed into teams of two humans and two robots versus two humans and two robots. The robots varied in anthropomorphism (anthropomorphic, mechanomorphic). Groups played a price-guessing game, with winners assigning noise blasts to all players. Then, participants completed surveys about their perceptions of the players. The results indicate how robot anthropomorphism moderates effects of group membership on survey and behavioral favoritism of ingroup and outgroup humans and robots. These results have moral implications: If participants are willing to give painful noise blasts to humans in order to spare their robot teammates, what else might they be willing to do?

## Related Work

People’s moral behavior and perceptions of others are affected by many factors. In this paper, I particularly focus on group membership, agent type, and robot anthropomorphism as factors relevant to humans-robot interaction (HRI), and I describe the motivation for this focus below.

### Group Membership Affects Anthropomorphism and Positive Responses

Group membership is critical to effective group interaction because people typically view the ingroup (i.e., their own group) more positively than the outgroup (i.e., other groups). People are more likely to cooperate with ingroup members (Tajfel et al., 1971; Turner et al., 1987), favor them morally (Leidner and Castano, 2012), and anthropomorphize them (i.e., humanize them; Haslam et al., 2008). This is a type of intergroup behavior.

Group interaction with robots often takes the form of intergroup behavior similar to social psychology (Fraune et al., 2015a, b, 2017a; Leite et al., 2015). Participants categorize robots as ingroup or outgroup members based on perceived robot gender (Eyssel et al., 2012), nationality (Kuchenbrandt et al., 2013; Correia et al., 2016), helpfulness (Bartneck et al., 2007), and robot use of group-based emotions (e.g., pride in the group; Correia et al., 2018).

The more strongly participants categorize robots as ingroup members, the more likely they are to perceive them as anthropomorphic (Kuchenbrandt et al., 2013) and give them more moral rights (Haslam et al., 2008; Kahn et al., 2011; Haslam and Loughnan, 2014). For example, when military squads socially bonded with bomb-defusing robots, they hesitated to send the robots into dangerous territories (Carpenter, 2016). Thus, robots’ group membership can affect moral decisions about them.

However, group effects in HRI do not directly reflect group effects in social psychology (Chang et al., 2012; Fraune and Šabanović, 2014; Fraune et al., 2019). Below, I examine a divergence in group-related responses toward humans and robots and some possible explanations.

#### People Differentiate More Within the Ingroup Than the Outgroup

In previous studies, participants differentiated between humans and robots to the extent that they showed different patterns in responses toward group members depending on agent type. Participants differentiated ingroup humans and robots more than outgroup humans and robots. Viewed another way, participants favored ingroup humans over outgroup humans more than they favored ingroup robots over outgroup robots (Fraune et al., 2020). Thus, the effect of group membership was stronger for humans than for robots. These findings can be viewed from the perspective of the outgroup homogeneity effect or from social identity theory, described below.

##### Outgroup homogeneity effect

In the outgroup homogeneity effect, output members are typically seen as more similar to each other, and ingroup members are typically seen as more diverse (Jones et al., 1981; Judd and Park, 1988; Ackerman et al., 2006). The outgroup homogeneity effect has been shown to occur in competitive contexts, even when there was no difference in the amount of information about exemplars of the ingroup and outgroup (Judd and Park, 1988). Thus, in previous studies, participants perceived more differences in ingroup members than outgroup members (Fraune et al., 2020), accounting for the differentiation between ingroup (but not outgroup) humans and robots.

##### Social identity theory

According to social identity theory (Turner and Tajfel, 1986), more prototypical group members have more influence over their group (Hogg, 2001) and experience more results of their group membership (Mastro and Kopacz, 2006). In prior studies, participants may have attended to differences between ingroup humans and robots and treated the ingroup robots as less prototypical of the ingroup (Van Knippenberg et al., 1994; Van Knippenberg, 2011). Ingroup robots being perceived as less prototypical ingroup members would account for findings that group effects from psychology extend to interaction with robots, but to a lesser degree than to interaction with humans.

In the prior study, the robots with which participants interacted were far from human. They had only minimally anthropomorphic features (e.g., head, eyes), were less than a foot tall, and had the shape of the upside down cup. This leads to the question, can manipulating how prototypical a robot is of a human group modify the extent to which robots experience the results of their group membership—that is, ingroup favoritism?

#### Agent Anthropomorphism Affects Prototypicality and Anthropomorphism

To modify how prototypical a robot group member is in relation to a human group, I selected anthropomorphism. The more anthropomorphic a robot is, the more readily it should fit into human groups. Anthropomorphism also confers other benefits: When people perceive agents as anthropomorphic, they typically behave morally toward them (Epley et al., 2007; Haslam et al., 2008; Waytz et al., 2010). For example, people usually consider it more important to behave morally toward humans than toward bugs. In other cases, when people dehumanize other humans, they treat those humans like they are animals (Haslam and Loughnan, 2014).

Considering others as similar or different from humans and treating them accordingly is typically divided into two factors: (1) Agents high in the ability to Experience emotions (e.g., warmth, fear, joy, suffering) are perceived as deserving more moral rights. People typically consider robots to have low experience (Gray et al., 2007; Wullenkord et al., 2016), leading them to indicate that robots deserve fewer moral rights than humans (Kahn et al., 2011; Lee and Lau, 2011). (2) Agents high in Agency (e.g., civility, rationality) are perceived as having high moral responsibility (Haslam et al., 2008). More complex robots are viewed as more agentic than simple robots (Kahn et al., 2011), but less agentic than adult humans (Gray et al., 2007). Thus, some robots could be perceived to have higher moral responsibility than others (Kahn et al., 2012).

**FIGURE 2:**
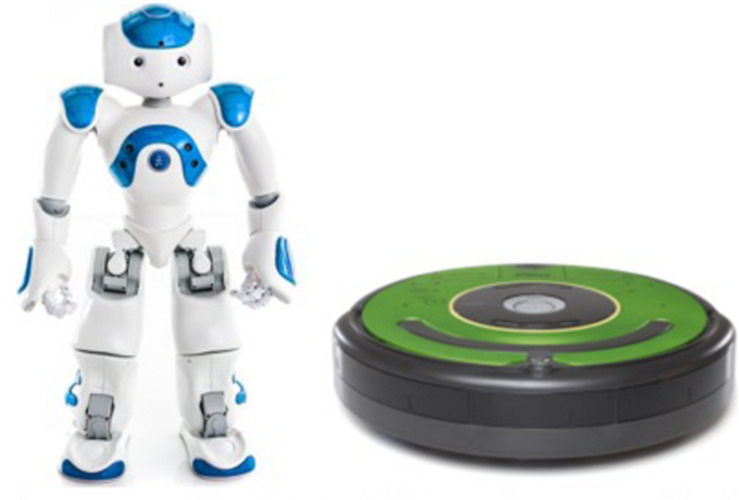
Anthropomorphic NAO **(left)**: mechanomorphic iRobot **(right)**.

In prior studies, participants treated robots as having less ability to experience than humans in ratings and by assigning them more loud and “painful” noise blasts (Fraune et al., 2017b, 2020). In this study, I specifically manipulated robot anthropomorphism. To do so, I used anthropomorphic and mechanomorphic robots that varied on appearance dimensions (Phillips et al., 2018), specifically: Body Manipulators (anthropomorphic robots had arms and a torso; mechanomorphic robots had only a circular body), Facial Features (anthropomorphic robots had a head, eyes, and a mouth; mechanomorphic did not), and Mechanical Locomotion (anthropomorphic robots had legs; mechanomorphic robots had wheels). I also manipulated robot behavior: anthropomorphic robots spoke English, and mechanomorphic robots only beeped (Figure 2). In this study, I purposely conflated robot appearance and behavior such that the anthropomorphic robots both looked and behaved in an anthropomorphic manner, and the mechanomorphic robots both looked and behaved in a mechanomorphic matter, as in former studies (Fraune et al., 2015b; de Visser et al., 2016). Researchers use this technique because mismatching form and behavior causes dissonance and reduces acceptance of robots (Goetz et al., 2003).

#### Ingroup Agents Are More Useful

Another difference between the ingroup and outgroup is that the ingroup is typically cooperative and useful to the team (Wildschut and Insko, 2007). Usefulness relates to more positive emotions and behavior the agent (Nass et al., 1996; Reeves and Nass, 1997; Bartneck et al., 2007; Saguy et al., 2015). Therefore, in this study, I measured perceived usefulness as a reason participants may treat ingroup robots favorably, even if they were mechanomorphic (Venkatesh et al., 2003).

### Present Study Overview

Overall, people treat robots somewhat, but not entirely, like humans in terms of ingroup favoritism. The more anthropomorphic the robot is, the more likely it should be that people will treat it as a prototypical group member and deserving of ingroup favoritism and moral status—but this has not yet been examined.

Previous studies measured moral behavior toward humans and robots by the volume of painful noise blasts participants assigned to others (Fraune et al., 2017b, 2020). Social psychological researchers have used noise blasts as a measure of aggression (e.g., Twenge et al., 2001). I specifically chose this measure because it violates the moral principle of harm (Leidner and Castano, 2012).

In this paper, I seek to replicate and extend the findings from previous studies. Below, I hypothesize about how people will treat robots. I define “treated better” on numerous measures, including being (a) treated more as part of participants’ group (e.g., rated as more cooperative and less competitive), (b) given softer noise blasts, (c) rated more positively on attitude valences and emotions, (d) anthropomorphized more, and (e) perceived as more useful.

First, I examine four hypotheses, replicating prior studies (Fraune et al., 2017b, 2020):

H1. Ingroup members will be treated better than outgroup members.
H2. Humans will be treated better than robots.
H3. The ingroup–outgroup difference will be larger than the human–robot difference, such that ingroup robots will be treated better than outgroup humans.
H4. Differences in ratings of ingroup humans and robots will be larger than differences in ratings of outgroup humans and robots.

Next, I test our main novel hypothesis from this study:

H5. Anthropomorphic robots will be differentiated less from humans than mechanomorphic robots from humans, across group memberships.

I examine if this relates to a consistent difference across robot anthropomorphism:

H6. Anthropomorphic robots will be treated better than Mechanomorphic robots.

I examine if prior findings that ingroup robots are treated better than outgroup humans (H4) depends on robot anthropomorphism:

H7. The ingroup–outgroup difference will be larger for anthropomorphic than mechanomorphic robots, such that the difference between ingroup anthropomorphic robots and outgroup humans will be greater than ingroup mechanomorphic robots and outgroup humans.

I examine in prior findings that the difference between ingroup humans and robots is greater than the difference between outgroup humans and robots (H5) depends on robot anthropomorphism:

H8: Differences in ratings of ingroup humans and mechanomorphic robots will be larger than differences of ingroup humans and anthropomorphic robots, which will be larger than differences in ratings of outgroup humans and robots.

Finally, I examine if group cohesion, anthropomorphism, and usefulness of agents relates to the volume of noise blasts participants give them.

H9: More perceived group cohesion, anthropomorphism, and usefulness will relate to lower noise blast volume.
H9a. Group cohesion will have the strongest effect for ingroup members.
H9b. Anthropomorphism will have the strongest effect for anthropomorphic robots.
H9c. Usefulness will have the strongest effect for mechanomorphic robots.

## Method

### Design

In this study, I use a 2 × 2 × 2 mixed design with Group Membership (ingroup, outgroup) and Agent Type (human, robot) manipulated within subjects’ and Robot Anthropomorphism (anthropomorphic, mechanomorphic) manipulated between subjects. The study was approved by the New Mexico State University Institutional Review Board (IRB).

### Participants

Participants were recruited through the psychology participant pool at New Mexico State University. The study contained 81 participants, divided per condition as Anthropomorphic: 45 (61.7% female) and Mechanomorphic: 36 (69.4% female). Participants were on average 19.15 years old, and the majority of participants indicated their race as White (66.3%). The other racial groups were Native American (3.6%), Asian (4.8%), Black (1.2%), or mixed race (12.0%). The majority also identified as Hispanic/Latino (68.7%).

### Procedure

Participants took part in the experiment in the Intergroup HRI lab (iHRI Lab) at New Mexico State University. The purpose was described as examining cognitive performance on a price-guessing game. Participants who objected to hearing loud noise blasts would be excused from the session; however, this never occurred.

When participants arrived at the study, they sat together at the table and, at the experimenter’s instruction, introduce themselves to each other by name. The experimenter randomly assigned participants to teams of two humans and two robots. Teammates received colored armbands related to their team (red or blue) and saw who was on their team. The experimenter told teams that they would work together on the task against the other team. The experimenter described the task to participant team members (see Task section) and then brought teams one at a time into the next room to meet their robot teammates (who wore the appropriately colored armbands on their bodies).

After meeting the robots, participants completed the task in separate rooms, and then the computer prompted participants to complete surveys. Finally, they were debriefed, given one credit for their psychology class, and dismissed.

### Robots

The robots differed depending on between-subject conditions (Figure 2). In the Anthropomorphic condition, two humanoid Nao robots greeted participants with human speech (e.g., “Hello, I’m Sam. I look forward to working with you”). These robots sat on a table near a computer so they were not far below human eye level. In the Mechanomorphic condition, iRobot Creates (with their “Clean” button covered) beeped at participants, and the experimenter told participants that they would be working with these robots. These robots sat on the ground, where they would typically functionally drive.

The experimenter asked human teammates to introduce themselves by name to the robots. Participants were told that, because these robots’ purpose included interaction with the real world, they hear in a similar way to humans, and that the noise blasts are comparably aversive to humans and robots. Then, the experimenter led participants to separate rooms for the task, so they had no more communication with other players.

### Task

Participants played a price-guessing game programmed using Java in Eclipse. A computer screen displayed an item (e.g., couch, watch), and participants guessed the price. They were told that teammates’ answers were averaged for a final answer. [This was to create teams in which the members were interdependent because prior research has indicated that interdependence is an important part of teams (e.g., Insko et al., 2013)]. The team that came the closest to the correct price on a given round won that round, and one member of the winning team was “randomly selected” to assign noise blasts of different levels to all eight players (including themselves) before the next round. The game included 20 rounds of the main game and one final round. For each round, participants saw the average guess for each team, the actual price, which team won, and if they were the player who would select the volume of noise blasts for this round.

In reality, the game was rigged such that participants actually played on their own, with other players’ responses simulated. Participants’ teams won 50% of the time and each participant was “randomly assigned” to give noise blasts four times.

In this study, teammate responses on the task were not attributed to an individual so participants could not learn which teammates behaved differently than they did and could not treat them differently based on behavior.

### Noise Blast Measure of Moral Behavior

After each round, one player assigned noise blasts to all eight players. The experimenter described the noise blast as just another part of the game. This was to avoid influencing participant use of the noise blast. In reality, the noise blast was used as a measure of moral behavior (i.e., violating the ethical principle of harm; Leidner and Castano, 2012), as in other studies (e.g., Fraune et al., 2017b, 2020; Twenge et al., 2001).

**FIGURE 3:**
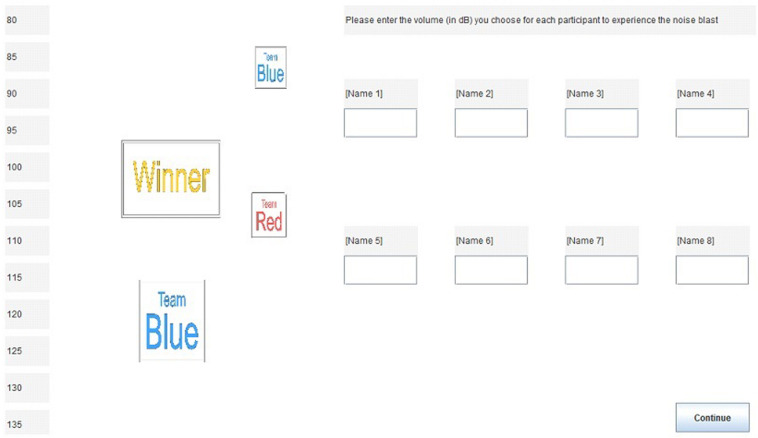
Screen for assigning noise blasts to Names 1–8 (actual participant and robot names were used during the experiment). Reproduced with permission from Fraune et al. (2017b).

The possible noise blasts were described as ranging in volume from 80 to 135 dB during all main rounds and from 110 to 165 dB during the final round, with 5-dB intervals. Each level of noise could be assigned to only one player to prevent participants from assigning everyone the same volume (e.g., to be fair; Figure 3). Each player (including the participant) was assigned one noise level per round. Participants viewed a chart relating different noise levels to known sounds (e.g., 80 dB = normal piano practice, 100 dB = piano fortissimo, 120 dB = threshold of pain, 135 dB = live rock band). In reality, participants never received noise blasts above 100 dB in order to protect their hearing. Also, the final round never arrived. Instead, participants were interrupted to complete surveys while they thought they were still playing the game. In this way, participants completed surveys while they were still part of a team with the robots and other humans.

During the noise blast phase, only the participant assigning noise volume could see what volumes were assigned. Other participants could not see who was assigning noise volume or what volume players received, only whether they won or lost that round. In doing so, participants could not be tempted to conform to the players’ behavior. In reality, the volumes for everyone other than the participant assigning the volumes were randomized by the computer program, but noise blasts from ingroup members were softer (0.85 times the outgroup noise blast) on average than the noise blast from outgroup members to simulate how teams often favor the ingroup. [In our previous study, participants delivered similarly softer (approximately 0.80 times) noise blasts to ingroup than outgroup members before they heard any noise blast from any other player (Fraune et al., 2017b)]. In a prior study, the difference in noise blast volume for ingroup than outgroup did not change the noise blasts participants gave to ingroup than outgroup members, as measured between before and after participants heard noise blasts assigned by others (Fraune et al., 2017b).

### Measures

**Noise blasts:** I used noise blast volume to measure moral behavior. I averaged noise blast volume to create one measure for each target (ingroup humans, ingroup robots, outgroup humans, outgroup robots). In a prior study, noise blast volume given to self and to the other ingroup human did not significantly differ (Fraune et al., 2017b).

**Surveys:** Participants rated surveys on a Likert scale, unless otherwise stated from 1 (Strongly Disagree) to 7 (Strongly Agree).

**Agents’ noise perceptions:** Participants responded to two questions (analyzed separately), indicating if they believed that the players “experienced pain from the noise blasts” and “did not like the noise” (Fraune et al., 2020).

**Group cohesion:** Participants responded to three questions (analyzed separately), indicating how much they felt cooperation and competition and as part of a group with players (Fraune et al., 2017b).

**Attitude valence and emotions:** Participants responded to questions (analyzed together) about their attitude valence toward robots on a bipolar scale from 1 (Dislike) to 7 (Like). They also rated how they felt on 12 emotions (e.g., happiness, fear) toward the players (Cottrell and Neuberg, 2005).

**Anthropomorphism:** To measure anthropomorphism, I examined agency (five items: can engage in a great deal of thought, has goals, is capable of doing things on purpose, is capable of planned action, is highly conscious) and experience (four items: can experience pain, can experience pleasure, has complex feelings, is capable of the motion; Kozak et al., 2006). Participants rated these on a scale from 1 (Not at all) to 4 (Average human) to 7 (Very much). I anchored ratings at “average human” to participants using shifting standards (Biernat and Manis, 1994) for rating humans than robots, as recommended in prior research (Fraune et al., 2017b, 2020).

**Usability:** Participants rated six questions about how useful it was to work with each player (e.g., “Working with this player in tasks like this would enable me to accomplish tasks more quickly”). These questions were modified from a prior scale (Venkatesh et al., 2003) to apply specifically to players in the game.

**Demographics:** Participants reported gender identity, age, major, and prior experience with robots and computers.

## Results

Data were analyzed in SPSS 25. Values of *p* < 0.050 were considered statistically significant and are reported below. All significant findings are reported.

I ran a series of 2 (Group Membership: ingroup/outgroup) × 2 (Agent Type: human/robot) × 2 (Robot Anthropomorphism: anthropomorphic/mechanomorphic) mixed ANOVAs, with the first two variables being within-participants and the last being between-participants. With these tests, I examined if:

H1. Ingroup members were treated better than outgroup members (main effect of group membership).
H2. Humans were treated better than robots (main effect of agent type).
H6. Anthropomorphic robots were treated better than Mechanomorphic robots (main effects of Anthropomorphism).

Some two-way interactions occurred. To examine these according to the hypotheses, I used 2 (Player: igR/ogH) × 2 (Robot Anthropomorphism anthropomorphic/mechanomorphic) ANOVAs to examine if:

H3. Ingroup robots were treated better than outgroup humans (main effect of player).
H7. The difference between ingroup anthropomorphic robots and outgroup humans was greater than ingroup mechanomorphic robots and outgroup humans (interaction effect).

I calculated ingroup Group Differentiation as the difference between ratings of ingroup humans and ingroup robots (igH-igR) and outgroup Group Differentiation as the difference between ratings of the outgroup humans and outgroup robot (ogH-ogR).

There has been contention over the use of difference scores, such as those calculated above (Peter et al., 1993; Edwards, 2001; Edwards and Schmitt, 2002). The main concerns are as follows: (1) For the construct examined, it may be that one of the variables should be weighted more than another, for which the method of difference scores cannot account and (2) the findings may not be replicable, which is partially because (3) measure reliability typically decreases from using difference scores compared to the reliability of each score individually (Peter et al., 1993). To address the first concern, I operationally define Group Differentiation as the linear difference between how people respond to the ingroup versus the outgroup, for each agent type. To address the second concerns, prior research has already shown the replicability of findings with this definition of Group Differentiation (Fraune et al., 2017b, 2020). To address the third concern, I report Cronbach’s alpha for the difference scores (denoted as α_diff_), all of which are very high (above 0.8), indicating that reliability is not a problem for different scores in this study. With these main concerns addressed, difference scores are appropriate in this context.

I used Group Differentiation (ingroup differentiation/outgroup differentiation) × 2 (Robot Anthropomorphism: anthropomorphic/mechanomorphic) ANOVAs to examine if:

H4. Differences in ratings of ingroup humans and ingroup robots were larger than differences in ratings of outgroup humans and outgroup robots (main effect of Group Differentiation).
H5. Mechanomorphic robots were differentiated more from humans than anthropomorphic robots from humans (main effects of Anthropomorphism).
H8: Differences in ratings of ingroup humans and mechanomorphic robots were larger than differences of ingroup humans and anthropomorphic robots, which were larger than differences in ratings of outgroup humans and robots (interaction effect).

I used *post hoc t*-tests to distinguish differences during interaction effects. Descriptive and inferential statistics are reported in tables and figures, and *post hoc* t-tests results are reported in the text.

Finally, I used linear regression to examine the effects of group cohesion, anthropomorphism, and usefulness on volume of noise blasts participants gave players. I examined this separately for ingroup than outgroup members, humans and robots, and anthropomorphic and mechanomorphic robots to determine if:

H9: More perceived group cohesion, anthropomorphism, and usefulness related to lower noise blast volume.
H9a. Group cohesion had the strongest effect for ingroup members.
H9b. Anthropomorphism had the strongest effect for anthropomorphic robots.
H9c. Usefulness had the strongest effect for mechanomorphic robots.

### Pain Check

Participants rated no differences in agents not liking the noise blasts or experiencing pain from them (Tables 1, 2). However, participants differentiated anthropomorphic robots less than mechanomorphic robots from humans on not liking the noise blasts and experiencing pain from them (H5). In fact, they rated mechanomorphic robots as experiencing less pain and less disliking of the noise blasts than humans, but anthropomorphic robots as experiencing more pain than and disliking the noise blasts even more than humans.

**TABLE 1:** Inferential statistics for pain and noise blasts, for all hypotheses.

	Not like noise blast			Pain			Noise blast		
			
	*F*	*p*	*n*_*p*_^2^	*F*	*p*	*n*_*p*_^2^	*F*	*p*	*n*_*p*_^2^
H1	1.01	0.319	–	0.63	0.431	–	113.06	<0.001	0.589
H2	0.92	0.341	–	2.38	0.127	–	17.46	<0.001	0.181
H3	0.00	0.984	–	2.87	0.094	–	24.33	<0.001	0.235
H4	0.32	0.575	–	2.14	0.147	–	0.02	0.880	–
H5	4.22	0.043	–	4.74	0.032	0.056	4.40	0.039	0.052
H6	0.21	0.647	–	0.10	0.758	–	2.81	0.098	–
**H7**	2.72	0.103	–	1.12	0.293	–	0.74	0.394	–
**H8**	0.55	0.462	–	0.00	0.975	–	2.75	0.101	–

**TABLE 2:** Ratings of ingroup (IG) and outgroup (OG) humans (H), and robots (R) that are anthropomorphic (A) and metamorphic (M) on pain.

	Not like noise blast			Pain		
		
	A	M	Total	A	M	Total
IG-H	4.21 (1.55)	4.21 (1.61)	4.21 (1.56)	3.40 (1.75)	3.71 (2.01)	3.53 (1.86)
IG-R	4.34 (1.67)	3.97 (1.87)	4.19 (1.75)	3.81 (1.85)	3.50 (1.83)	3.68 (1.84)
OG-H	3.98 (1.33)	4.32 (1.90)	4.12 (1.59)	3.17 (1.37)	3.35 (2.01)	3.25 (1.66)
OG-R	4.15 (1.59)	3.68 (1.65)	4.95 (1.62)	3.96 (1.83)	3.41 (1.76)	3.73 (1.81)

*Format: M(SD).*

### Noise Blast Volume

**FIGURE 4:**
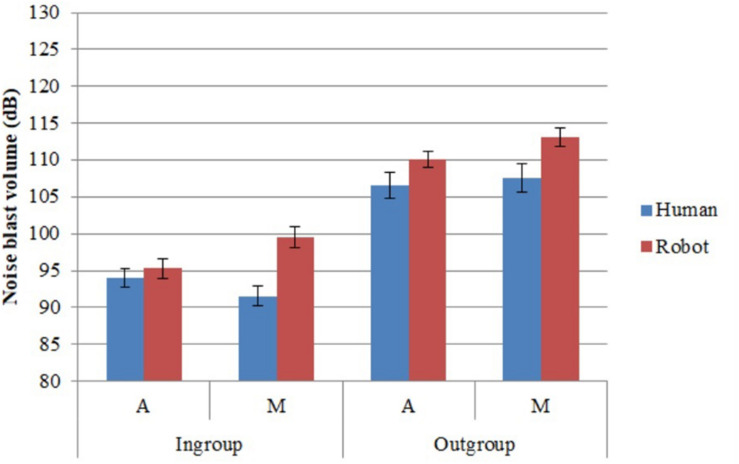
Noise blast volume selected for players (dB). Error bars represent standard error.

Participants assigned softer noise blasts to ingroup than outgroup members (H1) and humans than robots (H2; Table 1). They assigned softer noise blasts to ingroup robots than outgroup humans (H3). They differentiated anthropomorphic robots less than mechanomorphic robots from humans on noise blasts (H5; Figure 4).

### Attitude Valence and Emotions

Cronbach’s alphas was high for attitude valence (α = 0.984; α_diff_ = 0.983). Factor analysis indicated that, for each group (ingroup humans, ingroup robots, outgroup humans, outgroup robots) emotions were divided into two separate scales: Positive (respect, happiness, security, sympathy, excitement; α = 0.816; α_diff_ = 0.845) and Negative (discussed, fear, pity, anger, anxiety, sadness, unease; α = 953; α_diff_ = 0.895).

**FIGURE 5:**
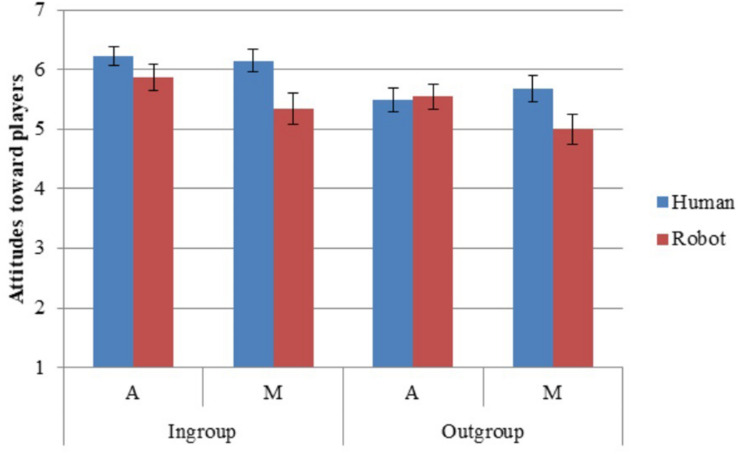
Attitudes toward players. Error bars represent standard error.

Participants had more positive attitude valence and fewer negative emotions toward the ingroup than outgroup (H1; Tables 3, 4). They had more positive attitudes and emotions toward humans than robots (H2). They showed more ingroup than outgroup differentiation in attitude valence—that is, ratings of humans as more positive than robots were more pronounced for attitudes toward the ingroup than the outgroup (H4). They differentiated anthropomorphic robots from humans less from mechanomorphic robots from humans on attitude valence and positive emotions (H5). Although there was no effect of Player (i.e., participants favoring ingroup robots over outgroup humans overall), there was an interaction effect between Player and Anthropomorphism on positive emotions (partly supporting H7; Table 3; Figure 5), with participants rating ingroup mechanomorphic robots as less positive than outgroup humans (mechanomorphic condition *p* = 0.044; anthropomorphic condition *p* = 0.047) and ingroup anthropomorphic robots (*p* = 0.022).

**TABLE 3:** Inferential statistics for attitude valence and emotions, for all hypotheses.

	Attitude valence			Positive emotion			Negative emotion		
			
	*F*	*p*	*n*_*p*_^2^	*F*	*p*	*n*_*p*_^2^	*F*	*p*	*n*_*p*_^2^
H1	17.34	<0.001	0.180	2.94	0.090	0.036	5.54	0.021	0.066
H2	10.55	0.002	0.118	15.86	<0.001	0.167	0.01	0.916	–
H3	0.01	0.924	–	2.86	0.095	–	2.43	0.123	–
H4	4.26	0.042	0.051	1.55	0.217	–	0.01	0.929	–
H5	5.20	0.025	0.061	8.22	0.005	0.093	0.12	0.676	–
H6	0.99	0.322	–	2.33	0.131	–	0.32	0.571	–
H7	3.99	0.049	–	5.77	0.019	0.068	0.23	0.635	–
H8	0.29	0.591	–	0.01	0.936	–	0.13	0.715	–

**TABLE 4:** Rating targets on attitude valence and emotions.

	Attitude valence			Positive emotions			Negative emotions		
			
	A	M	Total	A	M	Total	A	M	Total
IG-H	6.22 (1.05)	6.15 (1.15)	6.20 (1.09)	4.08 (1.01)	3.98 (1.28)	4.04 (1.12)	1.86 (1.24)	1.88 (1.17)	1.87 (1.20)
IG-R	5.86 (1.46)	5.34 (1.60)	5.64 (1.53)	3.81 (1.37)	3.05 (1.51)	3.49 (1.47)	1.87 (1.18)	1.91 (1.16)	1.89 (1.16)
OG-H	5.49 (1.35)	5.68 (1.30)	5.57 (1.32)	3.67 (1.25)	3.84 (1.31)	3.74 (1.27)	2.02 (1.18)	2.19 (1.51)	2.10 (1.32)
OG-R	5.54 (1.41)	5.00 (1.60)	5.32 (1.51)	3.63 (1.20)	3.04 (1.17)	3.38 (1.22)	1.96 (1.26)	2.26 (1.32)	2.09 (1.29)

*Format: M(SD).*

### Group Cohesion

**FIGURE 6:**
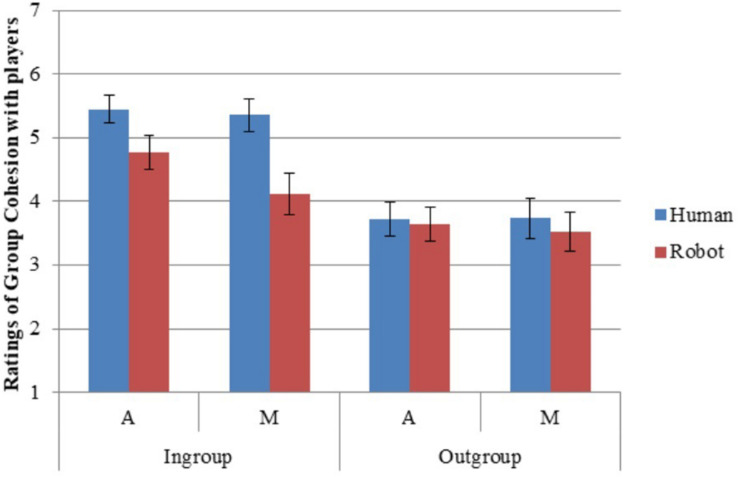
Group cohesion with players. Error bars represent standard error.

Participants indicated more feelings of group cohesion and cooperation, and less competition, with the ingroup than with the outgroup (H1; Tables 5, 6). They indicated more group cohesion and cooperation with humans than with robots (H2). They rated feeling more like part of the group and less competitive with ingroup robots than with outgroup humans (H3). They showed more ingroup than outgroup differentiation for group cohesion—that is, they indicated feeling more similar in cohesion between humans and robots in the ingroup than between humans and robots in the outgroup (H4; Figure 6).

**TABLE 5:** Inferential statistics for group cohesion, for all hypotheses.

	Group cohesion			Cooperation			Competition		
			
	*F*	*p*	*n*_*p*_^2^	*F*	*p*	*n*_*p*_^2^	*F*	*p*	*n*_*p*_^2^
H1	51.93	<0.001	0.397	31.19	<0.001	0.283	27.39	<0.001	0.257
H2	13.44	<0.001	0.145	16.57	<0.001	0.173	0.47	0.494	–
H3	8.03	0.006	0.092	0.87	0.353	–	19.53	<0.001	0.198
H4	10.69	0.002	0.118	0.28	0.096	–	3.51	0.065	0.042
H5	5.62	0.213	–	3.10	0.082	0.037	3.28	0.074	0.039
H6	3.47	0.472	–	0.00	0.964	–	0.61	0.438	–
H7	1.72	0.193	–	2.39	0.126	–	0.71	0.401	–
H8	1.72	0.193	–	0.98	0.324	–	0.48	0.491	–

**TABLE 6:** Ratings of players on group cohesion.

	Feel like a group			Cooperation			Competition		
			
	A	M	Total	A	M	Total	A	M	Total
IG-H	5.45 (1.36)	5.35 (1.57)	5.41 (1.57)	5.45 (1.49)	5.74 (1.46)	5.57 (1.47)	2.96 (1.82)	3.71 (2.18)	3.27 (1.00)
IG-R	4.77 (1.84)	4.12 (1.90)	4.49 (1.88)	4.96 (1.67)	4.50 (1.52)	4.77 (1.62)	3.53 (2.07)	3.44 (2.02)	3.49 (1.04)
OG-H	3.72 (1.70)	3.74 (2.05)	3.73 (1.84)	4.36 (1.67)	4.65 (1.72)	4.48 (1.69)	4.51 (1.79)	4.88 (1.77)	4.67 (1.78)
OG-R	3.63 (1.81)	3.53 (1.74)	3.59 (1.77)	4.04 (1.64)	3.97 (1.60)	4.01 (1.62)	4.34 (1.78)	4.24 (1.92)	4.30 (1.83)

*Format: M(SD).*

### Anthropomorphism

The experience subscale of anthropomorphism consisted of four items (α = 0.952; α_diff_ = 0.939), and the agency subscale included five items (α = 0.949; α_diff_ = 0.923).

Participants viewed humans as more experiential and agentic than robots (H2; Tables 7, 8). They also viewed ingroup robots as more experiential and agentic than outgroup humans (H3). There was more ingroup differentiation than outgroup differentiation for experience—that is, participants rated experience as more similar between humans and robots in the ingroup than between humans and robots in the outgroup (H4).

**TABLE 7:** Inferential statistics for anthropomorphism and usefulness, for all hypotheses.

	Experience			Agency			Useful		
			
	*F*	*p*	*n*_*p*_^2^	*F*	*p*	*n*_*p*_^2^	*F*	*p*	*n*_*p*_^2^
H1	0.00	0.954	–	0.06	0.813	–	8.84	0.004	1.010
H2	61.49	<0.001	0.438	53.27	<0.001	0.403	3.82	0.054	–
H3	41.95	<0.001	0.347	37.08	<0.001	0.319	0.05	0.821	–
H4	4.61	0.035	0.055	2.19	0.143	–	6.26	0.014	0.073
H5	2.32	0.131	–	0.95	0.333	–	4.22	0.043	0.051
H6	0.01	0.916	–	0.52	0.473	–	1.41	0.578	–
H7	0.30	0.086	–	0.99	0.322	–	4.48	0.038	0.054
H8	0.33	0.566	–	0.00	0.986	–	1.43	0.235	–

**TABLE 8:** Rating targets on agency and experience.

	Agency			Experience		
		
	A	M	Total	A	M	Total
IGH	5.86 (1.12)	5.52 (1.61)	5.72 (1.35)	5.75 (1.10)	5.54 (1.57)	5.66 (1.31)
IGR	4.21 (1.71)	4.24 (1.79)	4.22 (1.73)	3.51 (1.64)	3.90 (2.18)	3.67 (1.88)
OGH	5.71 (1.34)	5.32 (1.48)	5.54 (1.40)	5.69 (1.49)	5.15 (1.66)	5.46 (1.58)
OGR	5.71 (1.34)	4.31 (1.79)	4.36 (1.78)	3.82 (1.88)	4.06 (2.07)	3.92 (1.95)

*Format: M(SD).*

### Usefulness

**FIGURE 7:**
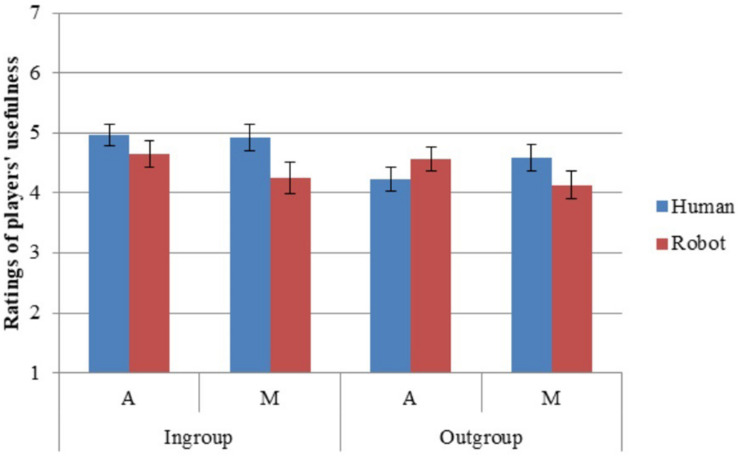
Ratings of player usefulness. Error bars represent standard error.

Cronbach’s alpha was high for the six usability items (α = 0.978; α_diff_ = 0.969). Participants rated ingroup members as more useful than outgroup members (H1; Tables 7, 8). They differentiated the ingroup more than the outgroup—that is, participants rated usefulness as more similar between humans and robots in the ingroup than between humans and robots in the outgroup (H4). They differentiated anthropomorphic robots from humans less than mechanomorphic robots from humans on usefulness (H5). They rated ingroup anthropomorphic robots as more useful than outgroup humans, but ingroup mechanomorphic robots as less useful than outgroup humans (partially supporting H7; Figure 7).

### Relationship Among Variables

To determine which variables (perceived group cohesion, anthropomorphism, usefulness) most strongly related to noise blast volume, I used Pearson correlations.

Results indicated that for ingroup humans and robots, increased perceptions of group cohesion (humans: *r* = −0.403, *p* < 0.001; robots: *r* = −0.260, *p* = 0.020; H9a) and usefulness (humans: *r* = −0.222, *p* = 0.048; robots: *r* = −0.330, *p* = 0.003; partially supporting H9c) related to decreased noise blasts. For ingroup robots, increased perceptions of agency (*r* = −0.252, *p* = 0.025; H9b) also related to decreased noise blasts. When robot type was divided by robot Anthropomorphism, only anthropomorphic robots had a correlation between usefulness (*r* = −0.325, *p* = 0.030) and noise blasts.

For outgroup humans and robots, no correlations occurred.

## Discussion

In this study, participants played a game with ingroup and outgroup humans and robots. The robots were either anthropomorphic (NAO) or mechanomorphic (iRobots). I measured how group membership, agent type, and robot anthropomorphism affected responses toward them. The results confirmed prior findings (H1–H4) and contributed novel findings (H5–H8). The results confirmed Hypotheses 1 and 2, with participants favoring the ingroup over the outgroup and humans over robots. Hypothesis 3 was partly supported, with participants typically favoring ingroup robots over outgroup humans. Hypothesis 4 was partly supported, with participants typically showing greater ingroup differentiation between humans and robots than outgroup differentiation between them. Novelly, I show that these effects are robust against robot anthropomorphism (H7 and H8 rejected). Also new, Hypothesis 5 was supported, with group effects of humans more closely mirrored by group effects of anthropomorphic robots than mechanomorphic robots. This finding did not relate to any consistent difference due to robot anthropomorphism (H6 rejected). Finally, if participants felt like other players were a cohesive part of the group or useful to the group, participants behaved more morally toward them—but only if they were ingroup members (H9 partly supported). These findings are described in more detail below.

Findings of participants favoring the ingroup (H1) and humans (H2) replicate the findings from previous studies (Fraune et al., 2017b, 2020). This is a robust finding. Favoring the ingroup occurred on the behavioral measure of noise blasts and on survey measures of attitude valence, emotion, group cohesion, and usefulness. Favoring humans occurred on behavioral measures of noise blasts and survey measures of attitude valence, emotion, group cohesion, and anthropomorphism.

This paper contributes the novel finding that group dynamics in human–human interaction are more closely mirrored by human interaction with anthropomorphic than mechanomorphic robots (H5). This occurred on behavioral measures of moral favoring and on survey measures of group cohesion, attitude valence, and usefulness. The findings did not merely reflect more positive responses toward anthropomorphic than mechanomorphic robots (H6 rejected). This implies that humans more readily apply group effects to robots that look and act more anthropomorphic—at least in brief interactions.

However, robot anthropomorphism was not strong enough in this study to mitigate favoring ingroup over outgroup (H7 rejected) or the outgroup homogeneity effect (H8 rejected). That is, even with mechanomorphic robots, participants treated ingroup robots better than outgroup humans (H3). Moreover, even with anthropomorphic robots, participants showed more ingroup than outgroup differentiation between humans and robots (H4). This indicates that these findings of ingroup favoring, and of ingroup differentiation between humans and robots, are robust across various robot types. However, ingroup differentiation may have decreased if the robots were less distinguishable from humans in appearance (e.g., Minato et al., 2004; Nishio et al., 2007) or had longer, more social interactions with participants before the task (Kahn et al., 2012).

Another novel finding from the study is that perceptions of group members, whether they were humans or robots, related to moral behavior (H9): The more participants perceived ingroup (but not outgroup) members as cohesive and useful, the softer the noise blasts participants assigned them. Further, the more participants perceived ingroup robots as anthropomorphic, the softer noise blasts participants assigned to them. This occurred regardless of robot anthropomorphism. These results align with findings from prior studies in social psychology of people favoring the ingroup and discriminating against the outgroup not out dislike for the outgroup, but because they feel close to the ingroup (Greenwald and Pettigrew, 2014).

Although this study showed some effects of robot anthropomorphism, there were not as many as hypothesized (H6, 7, and 8 rejected). This may seem surprising, considering that prior work suggests that people favor anthropomorphic robots over mechanomorphic robots (Gray et al., 2007). However, prior work shows that favoring of anthropomorphic robots depend on the number of robots (Fraune et al., 2015b) and context (Kuchenbrandt et al., 2011; Sauppé and Mutlu, 2015; Yogeeswaran et al., 2016) of interaction. In the context of this study, participants competed in a game and that competitive context was critical in the interaction. This is most strongly illustrated in the behavioral noise blast measure and the survey measure of group cohesion, which showed medium to large effect sizes for group membership (ingroup/outgroup) and only small effect sizes for agent type (human/robot). Given that participant behavior was only minimally affected by whether the target was human or robot and that people find it much more important to behave positively toward humans than robots (Epley et al., 2007; Haslam et al., 2008; Waytz et al., 2010), it follows that anthropomorphism had little significant effect. For other measures, like attitude, which had small effects for both group membership and agent type, it similarly follows that effects of robot type would be even more minimal.

Another possible reason for not finding many effects of robot anthropomorphism is that participants may have responded to the study’s mechanomorphic robot differently than usual because of the use of the iRobot Creates. iRobots may be familiar to participants because their bodies is the same as those of Roombas (typically meant for vacuuming). Research indicates that familiarity increases positive responses (Rindfleisch and Inman, 1998), even with robots (MacDorman, 2006). It is also possible that the robots’ typical purpose of cleaning affected participant responses negatively due to the mismatch of typical and current task. However, because the robots were not viewed more negatively than anthropomorphic robots, this is likely not the case.

This study does have some limitations. First, the findings apply best to short-term interactions with robots. In the long term, responses toward mechanomorphic robots may show stronger group effects. Second, although the sample size was large enough to find the main hypothesized effects, a larger sample size may have revealed more detailed three-way interaction effects and may have showed support for Hypotheses 7 and 8. However, with 81 viable participants in the study, if the effect had been at least moderate in size, it would likely have been revealed.

This study also acts as a foundation for future research. Prior work indicated that small differences in group composition of the teams (varying between one and three robots and humans in a team of four) did not affect findings in this situation (Fraune et al., 2020); however, recent research has indicated that larger changes in group composition affect some social phenomena such as conformity (Hertz et al., 2019). Future research should examine how larger differences in group composition affect moral behavior toward humans and robots.

Further routes for future examination include biological mechanisms for treating ingroup robots nearly as well as ingroup humans. For example, prior work indicates that oxytocin accounts for greater trust and compliance with automated agents (De Visser et al., 2017). Further, oxytocin is shown to motivate people for greater favoritism (De Dreu et al., 2011) and protection (De Dreu et al., 2012) of the ingroup. It remains to be seen if oxytocin related to group favoritism can account for treating ingroup robots more positively.

## Conclusion

In this study, participants played a game with ingroup and outgroup humans and robots—with robots being anthropomorphic or mechanomorphic. Participants favored the ingroup over the outgroup and humans over robots. The study provides the novel contribution that human group dynamics were more closely reflected by group dynamics with anthropomorphic than mechanomorphic robots. Further, the findings indicate that if participants felt like other players were a cohesive part of the group or useful to the group, participants behaved more morally toward them—but only if they were ingroup members. These results can inform future human–robot teaming about how people will likely treat robots in their teams depending on robot anthropomorphism.

## Data Availability Statement

The datasets generated for this study are available through the Open Science Framework (10.17605/OSF.IO/HCDNU).

## Ethics Statement

The studies involving human participants were reviewed and approved by New Mexico State University (NMSU) Institutional Review Board (IRB). The patients/participants provided their written informed consent to participate in this study.

## Author Contributions

MF contributed to the conceptualization, funding acquisition, methodology, project administration, supervision, formal analysis, and writing.

## Conflict of Interest

The author declares that the research was conducted in the absence of any commercial or financial relationships that could be construed as a potential conflict of interest.
